# Melatonin Restores Autophagic Flux by Activating the Sirt3/TFEB Signaling Pathway to Attenuate Doxorubicin-Induced Cardiomyopathy

**DOI:** 10.3390/antiox12091716

**Published:** 2023-09-04

**Authors:** Yanyan Ma, Jipeng Ma, Linhe Lu, Xiang Xiong, Yalan Shao, Jun Ren, Jian Yang, Jiankang Liu

**Affiliations:** 1 Center for Mitochondrial Biology and Medicine, The Key Laboratory of Biomedical Information Engineering of Ministry of Education, School of Life Science and Technology, Xi’an Jiaotong University, Xi’an 710049, China; 2 Department of Cardiovascular Surgery, Xijing Hospital, Air Force Medical University, Xi’an 710032, China; 3 Department of Cardiology, Shanghai Institute of Cardiovascular Diseases, Zhongshan Hospital, Fudan University, Shanghai 200032, China; 4 School of Health and Life Sciences, University of Health and Rehabilitation Sciences, Qingdao 266071, China

**Keywords:** melatonin, doxorubicin (DOX)-induced cardiomyopathy, autophagic flux, TFEB, Sirt3

## Abstract

Doxorubicin (DOX) chemotherapy in cancer patients increases the risk of the occurrence of cardiac dysfunction and even results in congestive heart failure. Despite the great progress of pathology in DOX-induced cardiomyopathy, the underlying molecular mechanisms remain elusive. Here, we investigate the protective effects and the underlying mechanisms of melatonin in DOX-induced cardiomyopathy. Our results clearly show that oral administration of melatonin prevented the deterioration of cardiac function caused by DOX treatment, which was evaluated by left ventricular ejection fraction and fractional shortening as well as cardiac fibrosis. The ejection fraction and fractional shortening in the DOX group were 49.48% and 25.5%, respectively, while melatonin treatment increased the ejection fraction and fractional shortening to 60.33 and 31.39 in wild-type mice. Cardiac fibrosis in the DOX group was 3.97%, while melatonin reduced cardiac fibrosis to 1.95% in wild-type mice. Sirt3 is a mitochondrial deacetylase and shows protective effects in diverse cardiovascular diseases. Therefore, to test whether Sirt3 is a key factor in protection, Sirt3 knockout mice were used, and it was found that the protective effects of melatonin in DOX-induced cardiomyopathy were partly abolished. Further analysis revealed that Sirt3 and its downstream molecule TFEB were downregulated in response to DOX treatment, while melatonin administration was able to significantly enhance the expressions of Sirt3 and TFEB. Our in vitro study demonstrated that melatonin enhanced lysosomal function by increasing the Sirt3-mediated increase at the TFEB level, and the accumulation of autolysosomes induced by DOX treatment was attenuated. Thus, autophagic flux disrupted by DOX treatment was restored by melatonin supplementation. In summary, our results demonstrate that melatonin protects the heart against DOX injury by the restoration of autophagic flux via the activation of the Sirt3/TFEB signaling pathway.

## 1. Introduction

Cardiomyopathies can result from gene mutation, obesity, diabetes, and drugs. The common forms in patients are dilated, hypertrophic, restrictive, and arrhythmogenic cardiomyopathy, most cases of which are attributed to over one thousand mutations in approximately 100 genes [1]. For example, mutations in filamin-C (*FLNC*) and αB-crystallin (*CRYAB*) cause familial restrictive cardiomyopathy, while novel mutations in desmin result in arrhythmogenic cardiomyopathy [2,3,4]. Doxorubicin (DOX) is an effective chemotherapeutic drug to treat a variety of human cancers, such as breast cancer, lymphomas, and gastric cancer [5,6,7]. However, the clinical use of this drug is restricted due to its cumulative dose-dependent cardiotoxicity, which often results in left ventricular dysfunction and even congestive heart failure, particularly in children [8,9].

Previous studies have revealed the underlying molecular mechanisms of DOX-induced cardiotoxicity, which include DNA damage [10], reactive oxygen species (ROS) generation [11], mitochondrial dysfunction [12], and cardiomyocyte apoptosis [13]. Specifically, autophagy was previously demonstrated to participate in the pathogenesis of DOX-induced cardiotoxicity [14,15,16,17]. As a conserved pathway in eukaryotic cells, autophagy can recycle damaged organelles and misfolded protein aggregates, thus playing an important role in maintaining cellular homeostasis and survival. Although the majority of studies have shown increased autophagy levels in response to DOX injury, contradictory results were observed in DOX-induced cardiotoxicity because of the diverse experimental settings as well as the complexity of autophagy [15,18,19,20,21].

Melatonin is a circulating hormone that is secreted by the pineal gland and plays a major role in the entrainment of circadian rhythms [22]. Recent studies have emphasized its protective role in diverse cardiac diseases, such as myocardial ischemia/reperfusion (I/R) injury, cardiac hypertrophy, and diabetic cardiomyopathy [23,24,25,26]. Melatonin was shown to scavenge ROS as an antioxidant to attenuate cardiac I/R injury via JAK2/STAT3 activation [25], to improve cardiac function via the upregulation of PGC-1β in pressure-overload-induced cardiac hypertrophy [23], and to protect hearts against DOX-induced myocardial injury [27,28,29]. Melatonin was also shown to mitigate DOX-induced cardiac injury by enhancing the cellular antioxidant capability, such as the activities of superoxide dismutase and glutathione peroxidase [27]. However, the precise molecular mechanisms of melatonin in DOX-induced cardiotoxicity remain largely unknown.

Sirt3 is a mitochondrial deacetylase and protects cardiac function via preserving mitochondrial function and enhancing cellular antioxidant activity in diverse pathological conditions [30,31,32]. It has been demonstrated that Sirt3 protects hearts from myocardial infarction by the upregulation of autophagy and improving mitochondrial biogenesis [33]. Sirt3 activation was shown to attenuate myocardial injury in diabetic cardiomyopathy by parkin-dependent mitophagy [34]. Although data have shown that Sirt3 attenuates mitochondrial dysfunction in doxorubicin-induced cardiotoxicity, whether Sirt3 is involved in melatonin’s protective role against DOX-induced cardiotoxicity has not been determined yet [35,36].

Therefore, in the present study, we employed Sirt3 knockout mice to elucidate the mechanism of melatonin in DOX-induced myocardial injury.

## 2. Materials and Methods

### 2.1. Animals and Experimental Protocols

All animal procedures were approved by the Animal Care and Use Committees at Air Force Medical University (Approval NO.: IACUC-20173030). The Sirt3 knockout (KO) mice used in this study were a gift from the Collaborative Innovation Center of Model Animal Wuhan University. The mice were fed with standard chow and tap water and housed under 22–24 °C. Male C57BL/6J mice and Sirt3 KO mice weighing over 20 g at the age of 8–10 weeks were used in this study.

The in vivo experiments with mice included 6 groups, as follows: The DOX (Sangon Biotech, Shanghai, China)-treated groups with SIRT3 KO mice (S3 KO-DOX group) and wild-type mice (WT-DOX group) received an intraperitoneal (i.p.) DOX injection (5 mg/kg body weight) three times every other day (cumulative amount 15 mg/kg body weight) and were then kept for 4 weeks after the last DOX injection; the SIRT3 KO mice (S3 KO group) and wild-type mice (WT group) in the control groups were injected i.p. with an equivalent volume of saline three times every other day and raised for another 4 weeks. The DOX plus melatonin (Weikeqi Biotech, Chengdu, China) groups with S3 KO mice (S3 KO-DOX-MT group) and wild-type mice (WT-DOX-MT group) received melatonin treatment from drinking water orally with a dosage of 20 mg/kg body weight/day for 6 weeks just 1 week before the first i.p. DOX injection, and then were i.p.-injected with DOX at a dose of 5 mg/kg body weight three times every other day (cumulative amount 15 mg/kg body weight). For these in vivo experiments, each group included 10–12 mice.

### 2.2. Echocardiography

The mice were lightly anesthetized with isoflurane in oxygen at day 28 (4 weeks) after the final DOX or saline injection. All hair in the thoracic region of the mice was removed using a depilatory cream, and they were placed on a heating pad to maintain their body temperature. To avoid the influence of different heart rates on cardiac function, the flow of isoflurane was adjusted from 1% to 2% to maintain their heart rates between 400 and 500 beats per minute. Two-dimensional and M-mode echocardiography was performed by using the Vevo 2100 high-resolution ultrasound imaging system (Visual Sonics, Toronto, ON, Canada) connected to a 40 MHz transducer probe. The images were captured and stored for further measurement. The left ventricular ejection fraction (LVEF) and left ventricular fractional shortening (LVFS) were calculated with Vevo 2100 Workstation software (Version 3.1) to evaluate cardiac function. After echocardiography, the mice were sacrificed and their hearts were collected for further experiments, such as Western blot analysis and histological study.

### 2.3. Histopathology

After euthanasia, the hearts were harvested and washed with phosphate-buffered saline (PBS) buffer. Then, cardiac tissue was fixed in 4% paraformaldehyde for 2 to 3 days, followed by paraffin embedding, and cut into 5 μm thick sections. To evaluate the degree of myocardial fibrosis, sections were stained with Masson’s trichrome. Briefly, three random fields at 200× magnification from each section were used for cardiac fibrosis detection. The myocardial fibrosis was quantified with ImageJ software (Version 1.31, NIH, Bethesda, MD, USA) and presented as a percentage of the blue-stained area normalized to the total area [37].

### 2.4. Cell Culture and Transduction of GFP-mRFP-LC3 Adenovirus

H9c2 cells were purchased from Shanghai Tiancheng Technology (Shanghai, China). They were routinely cultured in Dulbecco’s modified Eagle’s medium (DMEM; Hyclone, Logan, UT, USA) supplemented with 10% fetal bovine serum (FBS; Gibco, New York, NY, USA) and maintained at 37 °C in a humidified incubator containing 5% CO_2_. The cells were then digested using 0.25% trypsin-EDTA (Solarbio, Beijing, China) and subcultured at a ratio of 1:3 when reaching 70–80% confluence. They were then incubated with 1 μM DOX, 100 μM melatonin, or 1 μM DOX plus 100 μM melatonin in the indicated groups for 24 h, and then harvested for further analysis. If melatonin was used, the cells were pretreated with melatonin for 8 h before the addition of DOX.

For the fluorescent imaging, adenovirus encoding GFP-mRFP-LC3 (Hanbio, Shanghai, China) was used to assess the autophagic flux and distinguish the autolysosome from autophagosome. Upon reaching 50% confluence, the H9c2 cells were transduced with GFP-mRFP-LC3 adenovirus at a multiplicity of infection of 50 for 24 h. Then, they were treated with 1 μM DOX, 100 μM MT, or 1 μM DOX plus 100 μM MT in the indicated groups for 24 h. The transfected cells were observed, and the fluorescent images were obtained using an FV10i confocal microscope (Olympus, Tokyo, Japan). The excitation and emission wavelengths for green fluorescence and red fluorescence were 488 nm/507 nm and 532 nm/588 nm, respectively. The red dots only (red) and yellow dots (red/green) were counted separately and used for further analysis. 

### 2.5. siRNA Transfection Experiment

The rat Sirt3 siRNA was purchased from GenePharma (Shanghai, China). For the Sirt3 knockdown experiments, the H9c2 cells were transfected with rat Sirt3 siRNA using Lipofectamine 3000 (Invitrogen, Carlsbad, CA, USA) according to the manufacturer’s instructions for 24 h. Then, the cells were treated with 1 μM DOX in the presence or absence of 100 μM melatonin for 24 h, and prepared for further analysis. When melatonin was added, the cells were pretreated with melatonin for 8 h before the DOX treatment. The Scra siRNA was used as the negative control, and the sequence was as follows: 5′-UUCUCCGAACGUGUCACGUtt-3′, 5′-ACGUGACACGUUCGGAGAAtt-3′.

### 2.6. Western Blot

The cardiac tissues were homogenized in a radioimmunoprecipitation assay (RIPA) buffer (Beyotime, Shanghai, China) with a protease inhibitor cocktail (Roche, Indianapolis, IN, USA), and the total proteins were separated by 8–12% SDS-PAGE. Then, the proteins in the SDS-PAGE were electrotransferred to polyvinylidene fluoride (PVDF) membranes (Millipore, Billerica, MA, USA) for 1 to 1.5 h. The membranes were incubated with primary antibodies against Sirt3 (Cell Signaling Technology, Beverly, MA, USA), PGC-1α (Proteintech, Wuhan, Hubei, China), TFEB (Proteintech, Wuhan, Hubei, China), LAMP1 (Cell Signaling Technology, Beverly, MA, USA), LC3B (Cell Signaling Technology, Beverly, MA, USA), P62 (Cell Signaling Technology, Beverly, MA, USA), or GAPDH (cmcTAG, Milwaukee, WI, USA) overnight at a dilution of 1:1000 at 4 °C after blocking in 5% non-fat milk at room temperature for 2 h. The membranes were rinsed in 0.1% Tween 20 TBST buffer 5 times and probed with secondary antibodies conjugated with horseradish peroxidase at RT for 2 h. The membranes were imaged with an enhanced chemiluminescence detection system (Millipore, Billerica, MA, USA). The bands were visualized with a ChemiDoc Imaging System (Version 3.0, Biorad, Hercules, CA, USA), and the relative protein amounts were quantified using Image Lab software (Version 3.0, Biorad, Hercules, CA, USA). 

### 2.7. Measurement of Mechanical Properties of Cardiomyocytes 

Adult cardiomyocytes were isolated from the hearts by enzyme digestion, as previously described [38]. The cardiomyocytes were divided into five groups. 3-(1*H*-1,2,3-Triazol-4-yl)pyridine (3-TYP) is an inhibitor that suppresses the activity of Sirt3. By using this inhibitor, the role of Sirt3 in the mechanical properties of cardiomyocyte can be demonstrated following DOX treatment. The cells in the 3-TYP group were treated with 20 μM 3-TYP for 2 h, while the cells in the control group (CON group) were maintained without any treatment. The cells in the DOX group were incubated with 1 μM DOX for 30 min. The cells in the DOX-MT group were treated with 1 μM DOX for 30 min, followed by 100 μM melatonin for 2 h, whereas the cells in the DOX-MT-3-TYP group were treated with 1 μM DOX for 30 min, followed by 100 μM melatonin and 20 μM 3-TYP cotreatment for 2 h. 

The rod-shaped cardiomyocytes were obtained for an assessment of the mechanical properties after the diverse treatments. They were placed under an inverted microscope, and cell shortening was recorded with a SoftEdge MyoCam System (Version 6.5, IonOptix Corporation, Milton, MA, USA). The peak shortening, maximal velocity of shortening and relengthening (±dL/dt), time-to-peak shortening (TPS), and time-to-90% relengthening (TR90) were calculated to demonstrate the changes in the cardiomyocytes’ mechanical properties. 

### 2.8. Statistical Analysis

All the data are presented as the means ± SD and were analyzed using one-way ANOVA followed by a post-hoc Duncan’s test. A value of *p* < 0.05 was defined to be statistically significant. All the statistical tests were conducted with GraphPad Prism software version 5.0 (GraphPad Software, San Diego, CA, USA).

## 3. Results

### 3.1. Melatonin Administration Preserved Cardiac Function in DOX-Treated WT Mice but Not in Sirt3 KO Mice

**Figure 1:**
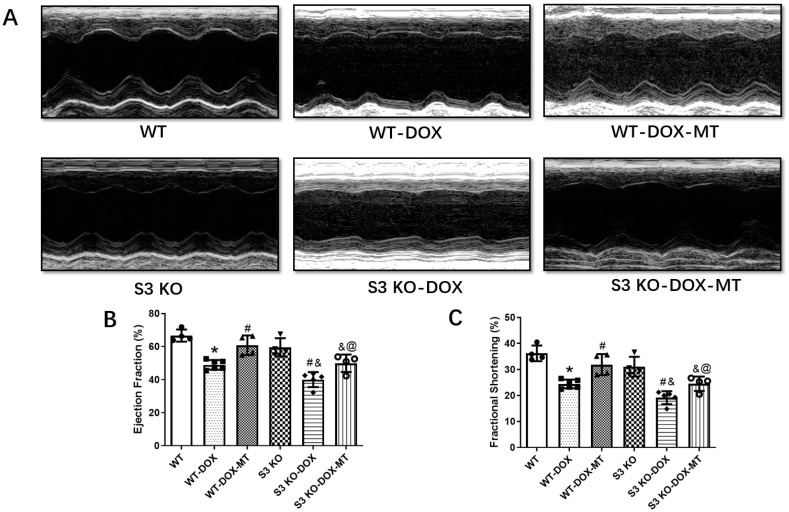
Echocardiography of melatonin with or without DOX treatment in S3 KO and wild-type mice. (**A**) Representative M-mode echocardiographic images of six respective mouse groups; (**B**) ejection fraction; (**C**) fractional shortening. WT, wild-type; DOX, doxorubicin; MT, melatonin; S3 KO, Sirt3 knockout. Mean ± SD, *n* = 4–6. * *p* < 0.05 vs. WT group, # *p* < 0.05 vs. WT-DOX group, & *p* < 0.05 vs. S3 KO group, @ *p* < 0.05 vs. WT-DOX-MT group.

The LVEF and LVFS assessed using echocardiography were significantly decreased in the DOX-treated WT (WT-DOX) mice compared to the WT mice without DOX treatment (Figure 1). Meanwhile, oral administration of melatonin significantly improved the cardiac function manifested by elevated LVEF and LVFS in the WT-DOX-MT group compared to the DOX-treated WT mice. However, DOX treatment further deteriorated the cardiac function in the *Sirt3* knockout mice compared to the DOX-treated WT mice. The LVEF and LVFS were slightly increased but were not significantly different in the S3 KO-DOX-MT group compared to the DOX-treated S3 KO mice. Furthermore, the LVEF and LVFS were improved in the WT-DOX-MT group compared to the mice in the S3 KO-DOX-MT group. These results demonstrate the important role of Sirt3 in mediating the protection of melatonin against DOX-induced cardiac dysfunction. 

### 3.2. Melatonin Treatment Failed to Alleviate Myocardial Fibrosis in DOX-Treated Sirt3 Knockout Mice

**Figure 2:**
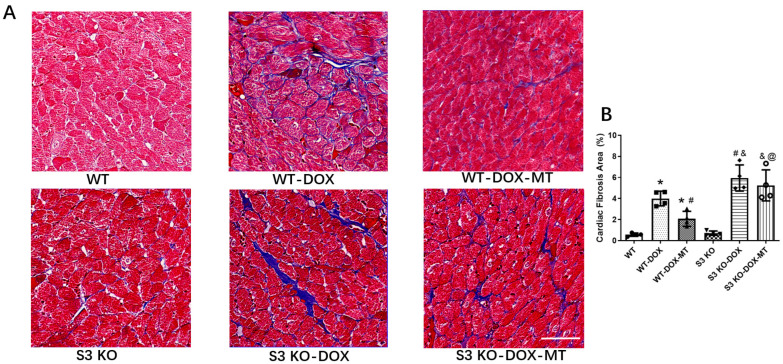
Cardiac fibrosis was assessed by Masson’s trichrome staining in S3 KO and WT mice following DOX and melatonin treatment. (**A**) Representative Masson’s trichrome staining images of heart sections; (**B**) analysis of interstitial fibrotic area of myocardial tissues. WT, wild-type; DOX, doxorubicin; MT, melatonin; S3 KO, Sirt3 knockout. Mean ± SD, *n* = 4 per group. * *p* < 0.05 vs. WT group, # *p* < 0.05 vs. WT-DOX group, & *p* < 0.05 vs. S3 KO-DOX-MT group, @ *p* < 0.05 vs. WT-DOX-MT group. Scale bar = 50 μm.

The cardiac fibrosis was assessed by quantifying the collagen deposition via Masson staining. The results shown in Figure 2 clearly reveal that chronic DOX treatment increased cardiac fibrosis compared to the WT mice, while the melatonin treatment reduced collagen accumulation compared to the mice in the WT-DOX group. DOX exacerbated cardiac fibrosis in the S3 KO mice compared to the DOX-treated WT mice. However, compared to the S3 KO-DOX mice, melatonin supplementation failed to reduce cardiac fibrosis in the S3 KO DOX-treated mice. Moreover, compared to the mice in the WT-DOX-MT group, myocardial fibrosis was further aggravated in the S3 KO-DOX-MT group. The data presented here illustrate that melatonin could attenuate cardiac fibrosis in WT mice but not in S3 KO mice in response to DOX treatment. 

### 3.3. Melatonin Mitigated Cardiac Dysfunction Induced by DOX by Upregulating Sirt3/TFE-Mediated Autophagic Flux

**Figure 3:**
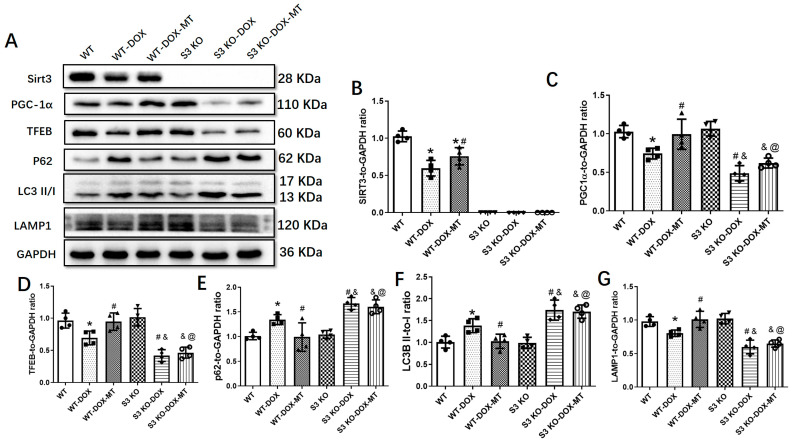
Effects of melatonin treatment on autophagic protein expressions in DOX-injured mice hearts. (**A**) Representative blot images of protein expressions in DOX-injured mouse hearts with or without melatonin treatment; (**B**) Sirt3 level; (**C**) PGC-1α level; (**D**) TFEB level; (**E**) P62 level; (**F**) LC3II/I ratio; and (**G**) LAMP1 level. WT, wild-type; DOX, doxorubicin; MT, melatonin; S3 KO, Sirt3 knockout. Mean ± SD, *n* = 4 per group. * *p* < 0.05 vs. WT group, # *p* < 0.05 vs. WT-DOX group, & *p* < 0.05 vs. S3 KO-DOX-MT group, @ *p* < 0.05 vs. WT-DOX-MT group.

We further investigated the role of the autophagy pathway mediated by Sirt3/TFEB. As shown in Figure 3, DOX treatment significantly upregulated the expressions of LC3B and P62 and decreased the Sirt3, PGC-1α, TFEB, and LAMP1 expressions, which were markedly reversed by melatonin administration in WT mice. However, melatonin showed no significant alterations in the expression levels of PGC-1α, TFEB, LAMP1, LC3B, and P62 in the DOX-treated S3 KO mice.

### 3.4. Melatonin Improved Cardiomyocyte Contractile Properties via the Sirt3 Signaling Pathway in DOX-Induced Cardiotoxicity

**Figure 4:**
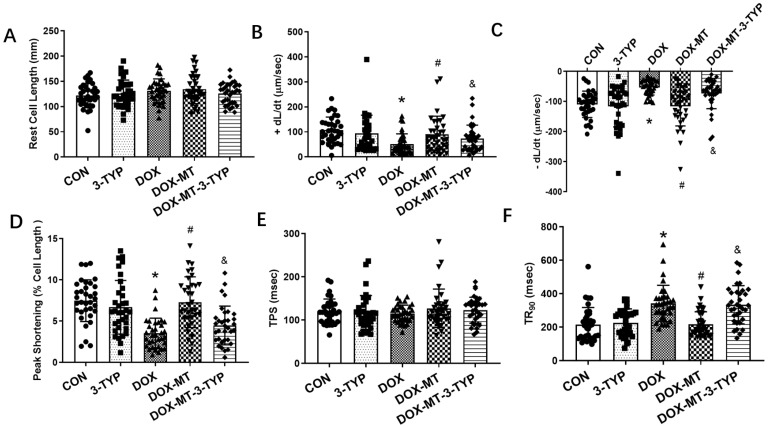
Contractile function of isolated cardiomyocytes with melatonin treatment and Sirt3 inhibitor 3-TYP. (**A**) Resting cell length; (**B**) maximal velocity of shortening (+dL/dt); (**C**) maximal velocity of relengthening (−dL/dt); (**D**) peak shortening (PS, normalized to resting cell length); (**E**) time-to-peak shortening (TPS); and (**F**) time-to-90% relengthening (TR_90_). Mean ± SD, *n* = 39 cells per group. * *p* < 0.05 vs. CON group, # *p* < 0.05 vs. DOX group, & *p* < 0.05 vs. DOX-MT group.

We further explored the role of the Sirt3 signaling pathway in the protection of melatonin against DOX-induced cardiomyocyte contractile dysfunction. The Sirt3 inhibitor, 3-TYP, was used to investigate the key role of Sirt3 in the cardiomyocyte contractile properties in vitro. The results in Figure 4 show that 3-TYP alone did not affect the indices of cardiomyocyte contractile properties compared to the CON group. However, compared to this group, DOX treatment significantly reduced the maximal velocity of shortening and relengthening and prolonged the time-to-90% relengthening, implying the impaired contractile dysfunction caused DOX treatment. Furthermore, melatonin treatment increased the maximal velocity of shortening and relengthening and shortened the time-to-90% relengthening compared to the DOX group, while 3-TYP treatment in the presence of DOX and melatonin was able to partially offset melatonin’s protection against the DOX-induced detrimental effects of cardiomyocyte mechanical properties manifested by the impaired maximal velocity of shortening and relengthening and the elevated time-to-90% relengthening.

### 3.5. Melatonin Improved the Autophagic Flux via Increasing the Sirt3 Expression in H9c2 Cells

**Figure 5:**
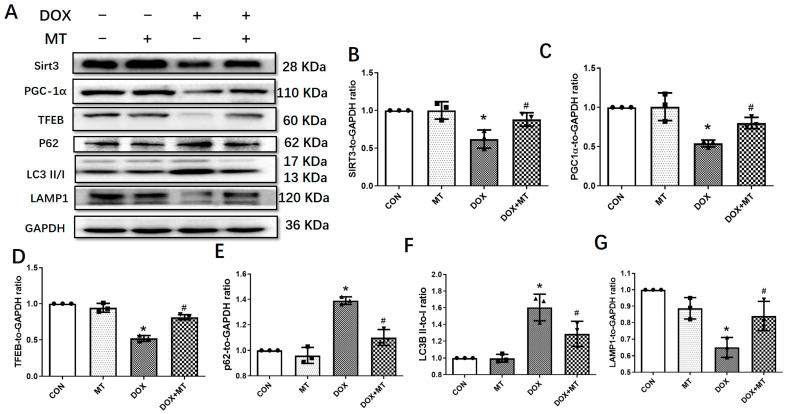
The effects of melatonin treatment on autophagic protein expressions in H9c2 myocytes treated with DOX. (**A**) Representative blot images of autophagic proteins with or without melatonin treatment; (**B**) Sirt3 level; (**C**) PGC-1α level; (**D**) TFEB level; (**E**) P62 level; (**F**) LC3II/I ratio; and (**G**) LAMP1 level. Mean ± SD, *n* = 3 per group. * *p* < 0.05 vs. CON group, # *p* < 0.05 vs. DOX group.

We further examined the role of melatonin in DOX-treated H9c2 cells. The data in Figure 5 show that the expressions of LC3B and P62 were increased, while the protein levels of Sirt3, PGC-1α, TFEB, and LAMP1 were downregulated following DOX treatment. Moreover, melatonin administration significantly reversed DOX-induced changes in the H9c2 cells. These data indicate that melatonin could activate the Sirt3/TFEB signaling pathway to protect H9c2 cardiomyocytes from DOX-induced cell toxicity.

**Figure 6:**
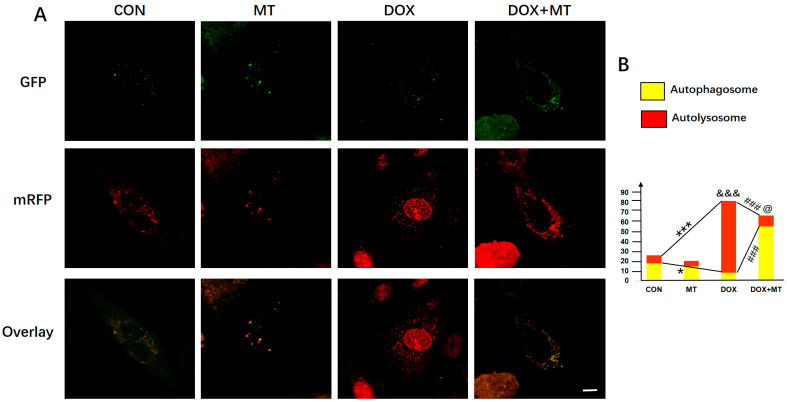
Evaluation of autophagic flux by ad-mRFP-GFP-LC3 infection with melatonin treatment in DOX-injured H9c2 cells. (**A**) Representative images of autophagosome and autolysosome in DOX-treated H9c2 cells with or without melatonin treatment; (**B**) autophagosome and autolysosome number in DOX-treated H9c2 cells with melatonin treatment; (**C**) autolysosome number in DOX-treated H9c2 cells with melatonin treatment. Scale bar = 10 μM, *n* = 10–15 cells per group. * *p* < 0.05 vs. CON group, ### *p* < 0.001 vs. DOX group. For total amount of autophagosomes and autolysosomes, &&& *p* < 0.001 vs. CON group, @ *p* < 0.05 vs. DOX group, *** *p* < 0.001 vs CON group.

To evaluate the autophagic flux, the ad-mRFP-GFP-LC3 adenovirus was transduced into the H9c2 cells to monitor the autophagosomes and autolysosomes, respectively, following the diverse treatments. Our data in Figure 6 show that DOX treatment markedly increased the number of autolysosomes and the total number of autophagosomes and autolysosomes compared to the CON group, while melatonin administration largely attenuated the DOX-induced accumulation of autolysosomes and reduced the total number of autophagosomes and autolysosomes, therefore restoring the autophagic flux. Because of the accumulation of autolysosomes in the DOX group, we speculate that the autophagic flux was blocked in response to DOX injury, while melatonin treatment may enhance the lysosomal function of decreasing the autolysosome number and restoring the autophagic flux.

### 3.6. The Restoration of Melatonin’s Effect on Autophagic Flux Was Abolished by Downregulating the Sirt3/TFEB Signaling Axis in DOX-Treated H9c2 Cells 

**Figure 7:**
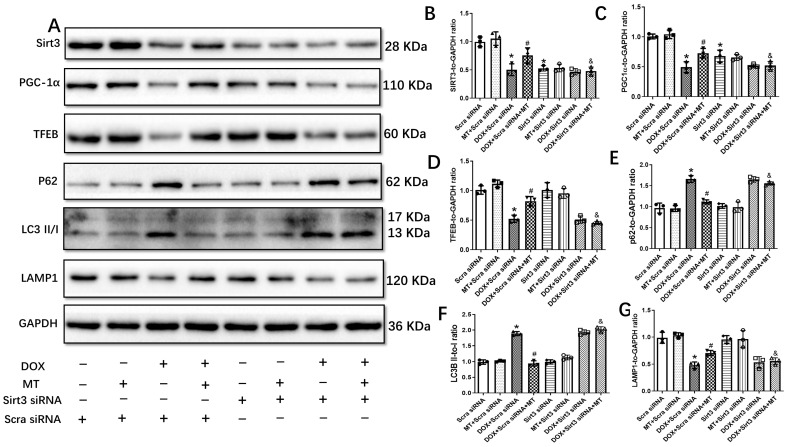
Autophagic protein expressions in DOX-treated H9c2 cells with Sirt3 siRNA under melatonin treatment. (**A**) Representative blot images of autophagic proteins with or without Sirt3 knockdown; (**B**) Sirt3 level; (**C**) PGC-1α level; (**D**) TFEB level; (**E**) P62 level; (**F**) LC3II/I ratio; and (**G**) LAMP1 level. Mean ± SD, *n* = 3 per group. * *p* < 0.05 vs. CON group, # *p* < 0.05 vs. DOX + Scra siRNA group, & *p* < 0.05 vs. DOX + Scra siRNA + MT group.

To verify the role of the Sirt3 signaling pathway in the DOX-induced inhibition of autophagic flux in H9c2 cells, Sirt3 siRNA was used to inhibit the expression of Sirt3. The results in Figure 7 reveal that Sirt3 knockdown significantly reduced the expressions of Sirt3, PGC-1α, TFEB, and LAMP1 following the DOX-plus-melatonin treatment compared to the DOX + Scra siRNA + MT group. Although melatonin administration restored the autophagic flux inhibited by DOX treatment, the downregulation of Sirt3 by Sirt3 siRNA abolished the effect of melatonin on autophagic flux in the H9c2 cells. 

## 4. Discussion

Numerous studies have demonstrated the protective roles of melatonin in diverse cardiac diseases, including cardiac I/R injury, myocardial infarction, and diabetic cardiomyopathy [24,26,39]. Melatonin is a potent antioxidant and plays an important role against cardiovascular diseases [40,41]. In one study, it impaired the phosphorylation of the p47*^phox^* subunit of nicotinamide adenine dinucleotide phosphate (NADPH) oxidase, disrupted its assembly, and further reduced reactive oxygen species production in Alzheimer’s disease [42]. Following peripheral nerve injury, melatonin treatment has also been shown to inhibit nicotinamide adenine dinucleotide phosphate-diaphorase and neuronal nitric oxide synthase expressions as an antioxidant to exert its neuroprotective effect [43]. As a key enzyme of the antioxidant system, nuclear factor erythroid 2-related factor 2 (Nrf2) has been shown to be regulated by melatonin. The activation of the Mst1-Nrf2 pathway has been shown to be protective against oxidative stress and apoptosis in cardiac hypertrophy and arginine vasopressin (AVP)-stressed cardiomyoblasts [44,45]. Furthermore, melatonin has been shown to alleviate DOX-induced cardiac injury by preserving mitochondrial membrane potential, reducing lipid peroxidation, and stimulating antioxidant activity [28,46,47,48]. However, the precise molecular mechanisms of melatonin against DOX-induced cardiotoxicity have not been fully elucidated. In the current study, we investigated and clarified the underlying molecular mechanisms involved in the protection of melatonin against DOX-induced cardiac injury. Our data show that DOX treatment markedly impaired cardiac function, assessed in terms of reduced LVEF and LVFS and increased cardiac fibrosis, which is consistent with previous studies [49,50]. Melatonin administration significantly elevated LVEF and LVFS; decreased cardiac fibrosis; regulated the levels of Sirt3, PGC-1α, TFEB, P62, LC3B, and LAMP1; and restored autophagic flux. However, these protective effects of melatonin were diminished in the DOX-treated S3 KO mice, which implies that the Sirt3 signaling pathway is a key player in mediating the protection of melatonin against DOX-induced cardiotoxicity. 

One of the most important findings from our current study is that melatonin attenuated DOX-induced cardiac injury by Sirt3 activation. Sirt3 is a mitochondrial deacetylase and plays an important role in maintaining cardiac function [33]. A previous study showed that Sirt3 protected hearts from cardiac I/R injury by regulating the activity of mitochondrial complex I and manganese superoxide dismutase (MnSOD) [51]. In a myocardial infarction model, Sirt3 attenuated cardiac injury post-myocardial infarction by improving microvascular function [52]. Furthermore, a couple of studies have emphasized the function of Sirt3 in attenuating cardiac hypertrophy by the activation of antioxidant enzymes, the suppression of ROS production, and the maintenance of mitochondrial function [32,53,54]. Importantly, it was reported that Sirt3 overexpression protected hearts from DOX-induced cardiotoxicity via mitigating mitochondrial DNA damage [35]. In another study, Sirt3 increased MnSOD expression and enhanced mitochondrial biogenesis in H9c2 cells treated with DOX [36]. In line with the data from previous studies, we showed that melatonin administration increased Sirt3 expression and protected hearts in a chronic DOX-induced cardiomyopathy model. However, DOX treatment aggravated cardiac function and fibrosis in Sirt3 KO mice compared to DOX-treated WT mice, which could not be rescued by melatonin administration. Together, these data illustrate that melatonin activates Sirt3 to protect hearts from DOX-induced cardiac injury.

Accumulating evidence indicates that autophagy participates in the pathogenesis of DOX-induced cardiomyopathy [55]. Autophagy is a multi-step cellular process that degrades misfolded proteins and damaged organelles, therefore maintaining cellular homeostasis [56]. It was previously reported that prior starvation restored cardiac autophagy via the activation of AMPK and ULK1 in response to acute DOX treatment [18]. Xu et al. [57] reported that macrophage migration inhibitory factor protected hearts from DOX-induced damage by promoting autolysosome formation. However, contradictory results were demonstrated in other in vivo and in vitro studies. For example, the inhibition of autophagy by aldehyde dehydrogenase 2 mitigated DOX-induced myocardial dysfunction in a chronic mouse model [15]. Furthermore, in another study, it was shown that GATA4 reduced DOX-induced cardiomyocyte toxicity via suppressing excessive autophagy [14]. Since the observation of any single protein involved in the autophagy pathway may not display the whole picture of autophagy, we speculate that these contradictory data may be due to the complexity of this process as well as the dosage of DOX used in the experiments. In this study, we found that Sirt3 expression activated by melatonin treatment increased PGC-1α expression. However, the mechanism by which Sirt3 regulates PGC-1α is still elusive. Through immunoprecipitation, a direct interaction between Sirt3 and PGC-1α was not observed in our results. Consistent with our results, previous studies have also shown that Sirt3 could increase PGC-1α expression [58,59]. As a master regulator of lysosome biogenesis, the increased TFEB expression enhanced LAMP1 expression, thus improving lysosomal function. The DOX-induced disruption of autophagic flux was ultimately restored by melatonin treatment. In line with these data, our results show that DOX treatment significantly increased the number of autolysosomes in the H9c2 cells by using GFP-mRFP-LC3 adenovirus. Interestingly, a recent study also showed that DOX disturbed autophagic flux due to lysosome dysfunction, while the inhibition of autophagy initiation by Beclin1 haploinsufficiency attenuated DOX-induced cardiac injury [16]. Our data reveal that melatonin treatment greatly reduced the number of autolysosomes, which implies that the DOX-induced inhibition of autolysosomal degradation was partly attenuated by melatonin treatment. Further analysis showed that the protective role of melatonin against DOX-induced cardiotoxicity was mediated by increasing TFEB expression, and therefore, enhancing lysosomal function. Taken together, our results demonstrate that DOX significantly impaired cardiac function due to the inhibition of autolysosomal degradation, while melatonin administration improved lysosomal function and restored autophagic flux. 

## 5. Conclusions

In summary, the present study reveals that melatonin treatment significantly improves heart cardiac function and slows cardiac fibrosis from DOX-induced cardiomyopathy by activating the Sirt3/TFEB axis. These results may provide potential therapeutic targets for the clinical study of patients with DOX-induced cardiac injury. 

## Data Availability

Data are contained within the article.
